# Yeast Mannan-Rich Fraction Modulates Endogenous Reactive Oxygen Species Generation and Antibiotic Sensitivity in Resistant *E. coli*

**DOI:** 10.3390/ijms24010218

**Published:** 2022-12-22

**Authors:** Helen Smith, Sharon Grant, Paula Meleady, Michael Henry, Donal O’Gorman, Martin Clynes, Richard Murphy

**Affiliations:** 1 Alltech, Summerhill Road, Dunboyne, A86X006 Meath, Ireland; 2 National Institute for Cellular Biotechnology, Dublin City University (DCU), D09NR58 Dublin, Ireland

**Keywords:** antimicrobial resistance, yeast mannan fraction, bacterial metabolism, antibiotic susceptibility, reactive oxygen species

## Abstract

Mannan-rich fraction (MRF) isolated from *Saccharomyces cerevisiae* has been studied for its beneficial impact on animal intestinal health. Herein, we examined how MRF affected the formation of reactive oxygen species (ROS), impacting antibiotic susceptibility in resistant *Escherichia coli* through the modulation of bacterial metabolism. The role of MRF in effecting proteomic change was examined using a proteomics-based approach. The results showed that MRF, when combined with bactericidal antibiotic treatment, increased ROS production in resistant *E. coli* by 59.29 ± 4.03% compared to the control (*p* ≤ 0.05). We further examined the effect of MRF alone and in combination with antibiotic treatment on *E. coli* growth and explored how MRF potentiates bacterial susceptibility to antibiotics via proteomic changes in key metabolic pathways. Herein we demonstrated that MRF supplementation in the growth media of ampicillin-resistant *E. coli* had a significant impact on the normal translational control of the central metabolic pathways, including those involved in the glycolysis–TCA cycle (*p* ≤ 0.05).

## 1. Introduction

More than ever, alternative strategies are required to further reduce on-farm antibiotic use while supporting the restriction of antibiotics in animals to therapeutic use. Dietary mannan-oligosaccharide (MOS) prebiotics have been extensively studied in animal nutrition for their ability to bind and limit the colonization of intestinal pathogens [1,2,3,4,5,6]. Further refinements of yeast MOS have led to the isolation of a mannan-rich fraction (MRF) with enhanced benefits for intestinal health [7]. Recent research has demonstrated enhanced antibiotic susceptibility in *E. coli* following MRF supplementation via cellular metabolic and energetic modulation [8]. Cellular energetics and oxidative stress response are impacted by bacteriostatic and bactericidal antibiotics, affecting metabolism, energy output, and the production of reactive oxygen species (ROS) [9,10,11]. Belenky et al. [9] highlighted that metabolic changes following antibiotic interaction induced cytotoxic cellular damage. Research has also shown that the expression of the central metabolic pathways may be modified by altering the genes involved in metabolism [12,13,14,15]. As such, intentional metabolic modulation has been studied for its ability to produce molecules of potential biotechnological value [16]. Herein, we explored the impact of MRF derived from dietary prebiotic MOS on metabolic modulation via ROS generation and its relationship with antibiotic treatment in *E. coli* resistant to antibiotics.

*E. coli*, a facultative heterotroph, metabolizes glucose through the reactions of glycolysis, pyruvate decarboxylation, and the tricarboxylic acid cycle (TCA), critical for the aerobic growth of the bacterium [17,18]. The TCA cycle has long been considered a “housekeeping” pathway in *E. coli*, being highly regulated at the transcriptional level, with much of this control exerted in response to respiratory conditions [19]. Elements of bacterial metabolic networks such as components of the electron transport chain, amino acid metabolism, fatty acids, or nucleotides, as well as elements that play a primary role in microbial physiology, are believed to modulate the intrinsic resistome of bacterial pathogens, indicating that there exists a linkage between bacterial metabolism and bacterial susceptibility to antibiotics [20].

Bacteria have developed complex, adapted gene regulatory responses to oxidative stress, perhaps due to the prevalence of ROS produced endogenously through metabolism [21]. The formation of hydroxyl radicals leading to cell death is based on a common mechanism involving the transient depletion of NADH, the leaching of iron from iron-sulfur clusters, and the stimulation of the Fenton reaction [11]. Metabolic changes caused by bactericidal antibiotics such as β-lactams induce a complex set of metabolic changes in bacteria downstream of their direct target interaction, generating toxic by-products such as ROS mediated via TCA [10,11,22].

In this study, we examined the translational changes resultant from MRF addition to the growth medium of antibiotic-resistant *E. coli*. Of interest were the metabolic pathways and enzyme complexes directly affected by TCA–glycolysis, which impacts NAD(H) production, and as such the cellular energetic state and energy output. Previous research demonstrated the effect of yeast MRF on the cellular energetic state, effecting growth and phenotypic resistance to antimicrobial agents [8]. To further characterize the impact of the prebiotic material on antimicrobial resistance patterns, this study explored the effect of MRF on functional metabolic pathways, describing the translational change within the resistant model organism and its impact on ROS production as a potential contributing factor to enhanced antimicrobial susceptibility.

## 2. Results

### 2.1. MRF-Mediated ROS Production Enhances Antibiotic Activity against E. coli

**Figure 1:**
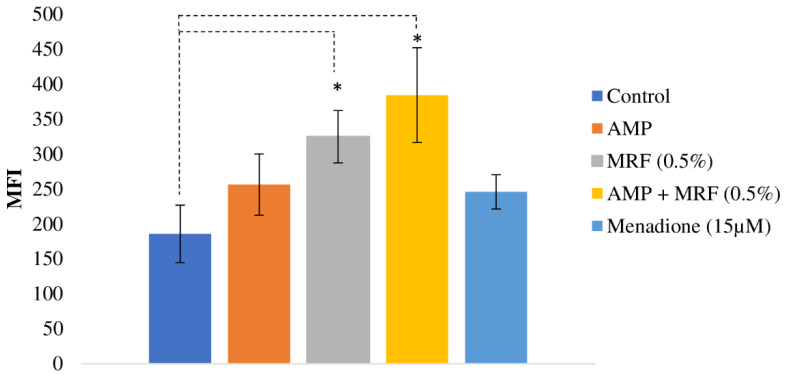
The effect of MRF on ROS production in antibiotic-resistant *E. coli* with and without antibiotic treatment. Diagram displays mean fluorescent intensity (MFI/OD595nm) of antibiotic-resistant *E. coli.* Antibiotic-resistant *E. coli* treated with 5 mgmL^−1^ ampicillin (AMP). Standard deviation is represented by error bars (*n* = 6). Means that were significantly different to the control are marked with an asterisk (*) (*p* ≤ 0.01, ANOVA, Fisher-LSD).

ROS have emerged as having a significant role in microbial metabolism and response to environmental stressors such as antibiotic exposure [23]. Previous research demonstrated that supplementation with MRF accelerated bacterial respiration in *E. coli* resistant to antibiotics [8]. Understanding that ROS are generated by aerobic respiration, we investigated whether MRF also impacts ROS production, potentially contributing to subsequent antibiotic-mediated impacts on *E. coli*. The Fluorometric Intracellular ROS assay kit allows the sensitive, one-step fluorometric detection of intracellular ROS (superoxide and hydroxyl radicals). MRF supplementation in the growth media of antibiotic-resistant *E. coli* cultures resulted in higher levels of ROS (42.34 ± 14.8%) compared to the control (*p* ≤ 0.05) (Figure 1). When MRF was combined with bactericidal antibiotic treatment, intracellular ROS production in resistant *E. coli* was 59.29 ± 4.03 greater than the control (*p* ≤ 0.05) (Figure 1), demonstrating an adjuvant response to combined MRF and antibiotic treatment. The further examination of resistant *E. coli* demonstrated that the amount of ROS generated from the combinatorial treatment (AMP + MRF) was 43.39% ± 2.82% greater than that for the treatment with ampicillin alone (Figure 1). The effect of increasing the MRF concentration, as well as the prolonged MFI of each treatment over time, can be examined in greater detail in the supplementary information (Figures S1 and S2, respectively).

Following bactericidal antibiotic treatment, susceptible bacteria succumb to damage to their proteins, membrane lipids, and DNA caused by hydroxyl radical formation [11]. However, antibiotic resistance modulates this response. Enhanced ROS generation is believed to initiate a damage response system to ROS within the organism [24,25]. Previous research has shown that amplification of endogenous ROS production via the impairment of the crucial protective biological response may increase sensitivity to hydroxyl radical-induced DNA damage/oxidative attack [26]. In addition, despite the harmfulness of ROS, moderate oxidative stress may have a positive effect on bacterial adaptability to antibiotics [27]. The resistant organism stimulated greater ROS production when supplemented with MRF, potentiating the link between endogenous metabolic perturbations, ROS production, and the modulation of the antibiotic susceptibility of the resistant organism.

### 2.2. MRF Effects Translational Change in the TCA Cycle 

To further our investigation, we assessed the translation of the central metabolic pathways of the resistant organism for changes following MRF and/or antibiotic treatment. Several central metabolic pathways controlled through enzyme activity and transcription are known to be affected by translational changes [28]. The proteomic basis for the observed physiological changes noted previously [8] focused on the glycolysis–TCA cycle and briefly explored other central metabolic pathways (pentose phosphate pathway, EMP pathway, acetate metabolism, and oxidative phosphorylation).

Research has shown that following bactericidal treatment, enhanced TCA cycling produces a burst in superoxide generation via respiration [11]. Such an event is capable of destabilizing iron–sulfur clusters, further stimulating the Fenton reaction, and causing cell death [11]. It was hypothesized that greater superoxide generation, as observed in Figure 1, may be resultant of a translational increase in TCA-cycle protein expression, potentiating antibiotic susceptibility following exposure to bactericidal drugs and/or exogenous MRF supplementation. Translational changes relating to the citric acid cycle (TCA) in response to MRF are noted in Table 1.

**Figure 2:**
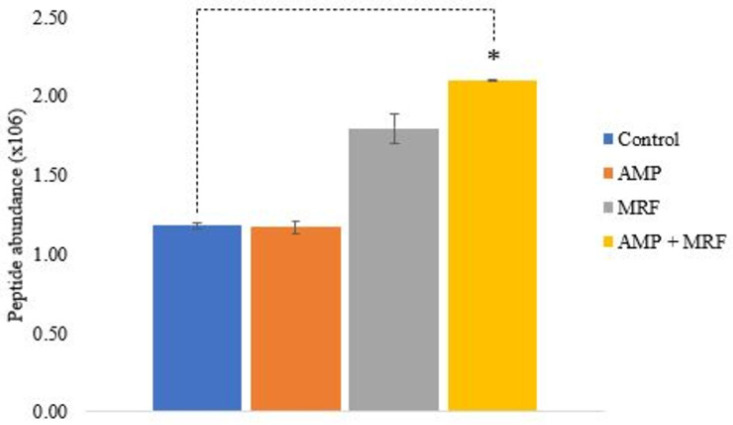
Normalized peptide abundance of *E. coli* regulatory protein ARCA. Control culture treated with AMP (0.1 mgmL^−1^ AMP), MRF (1 mgmL^−1^), or a combination of AMP (0.1 mgmL^−1^) + MRF (1 mgmL^−1^). Standard error is represented by error bars. Means that were significantly different to control are marked with an asterisk (*); *p* ≤ 0.03, ANOVA, T-test, two-sample assuming unequal variances.

In this study, the proteomic analysis of antibiotic-resistant *E. coli* revealed translational changes mediated by exogenous supplementation with MRF. Aerobic respiration control protein (ARCA), a cytoplasmic response regulator, is known to greatly influence central metabolism [16]; additionally, research has demonstrated that metabolic changes responsible for greater ROS production are induced by ARCA [10,29]. Notably, the expression of ARCA was significantly higher in *E. coli* cultures supplemented with MRF, with maximum peptide abundance noted when supplemental MRF was combined with antibiotic treatment (*p* ≤ 0.03) (Table 1 and Figure 2). Following combined MRF supplementation and ampicillin treatment, the expression of isocitrate dehydrogenase (ICD) and citrate synthase (GLTA), both involved in the control of flux in the TCA cycle, was elevated 0.72- and 1.65-fold compared to control conditions, respectively (*p* ≤ 0.05) (Table 1). Aconitase A (ACNA) and aconitase B (ACNB) are the two main forms of aconitase in *E. coli*; ACNB functions as the main catabolic enzyme in the TCA cycle, and ACNA responds to oxidative stress [11]. Relevant research has shown the absence of ACNB, or ICD-induced quinolone resistance, demonstrating the significance of specific central genes encoding functional proteins in phenotypic resistance [20,30,31]. Herein, exogenous supplementation with MRF combined with antibiotic treatment resulted in significantly higher protein expression of ARCA, ICD, GLTA, and ACNB, resulting in the greatest max fold change under these growth conditions (*p* ≤ 0.05) (Table 1, Figure 2).

**Figure 3:**
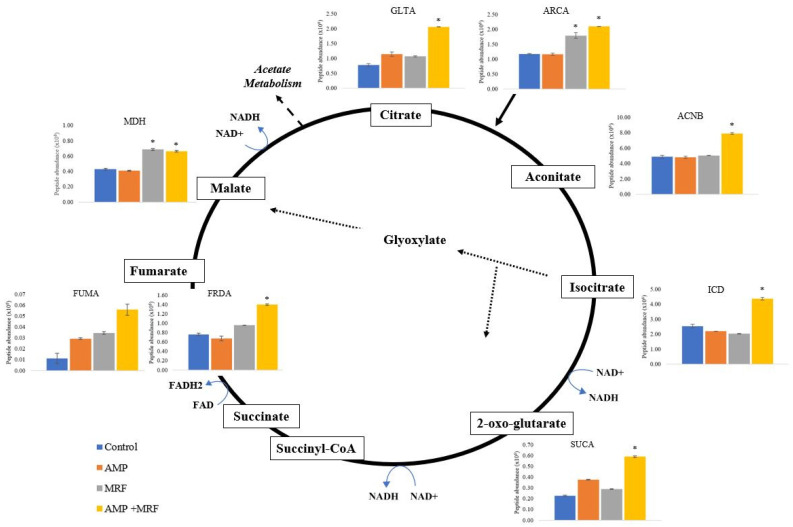
The influence of MRF on the translation of key intermediates of the citric acid cycle. The figure illustrates a comparison of normalized peptide abundance of control, AMP (0.1 mgmL^−1^ AMP), MRF (1 mgmL^−1^), and a combination of AMP (0.1 mgmL^−1^) + MRF (1 mgmL^−1^) cultures. Standard error is represented by error bars. Means that were significantly different to control are marked with an asterisk (*); *p* ≤ 0.05, ANOVA. Abbreviations: GLTA, citrate synthase; ARCA, aerobic respiration control protein; ACNB, aconitate hydratase; ICD, isocitrate dehydrogenase (NADP); SUCA, 2-oxoglutarate dehydrogenase E1 component; FUMA, fumarate hydratase class I; FRDA, fumarate reductase flavoprotein subunit; MDH, malate dehydrogenase.

Figure 3 illustrates the upregulation of key TCA-cycle proteins of the resistant model organism following exogenous MRF supplementation with and without ampicillin treatment. ACNAB, ICD, SUCA, and MDH expression were higher following MRF supplementation, which potentiates the glyoxylate shunt (GS)/bypass of the metabolic pathway (Table 1 and Table S1 and Figure 3). The GS bypasses the decarboxylation steps of the TCA cycle by which NADH is produced, and is upregulated under conditions of oxidative stress, antibiotic stress, and host infection [32]. According to Ahn et al. [32], by upregulating this shunt, bacteria generate less NADH to avoid antibiotic-induced ROS production. However, our data revealed that MRF and MRF in combination with antibiotic treatment had a significant impact on functional TCA-cycle proteins, which may have consequently influenced the GS shunt and NADH generation of the resistant organism. This may have been a response to the induction of oxidative or antibiotic stress, or the overload of NADH compared to the control culture conditions. Nonetheless, it is theorized that the GS generates greater tolerability or stress adaptation to oxidative stress [10,32]. The tested conditions highlighted a significant impact on the translation of central metabolic intermediates, with notable increased peptide abundance following MRF supplementation either alone or combined with bactericidal antibiotic treatment, yielding potential downstream consequences relating to oxidative phosphorylation and the induction of oxidative stress with physiological significance.

In previous relevant research, changes of a phenotypic nature related to TCA-cycle knockouts demonstrated the link between metabolism and efficient bactericidal killing [11]. This observation is consistent with carbon source limitation restricting bactericidal activity [11,33]. In this study, we propose that exogenous supplementation with MRF may potentiate the efficiency of bactericidal antibiotics via hydroxyl radical formation resulting from TCA NADH generation. Yeast MRF is capable of stimulating hydroxyl radical formation in resistant *E. coli* by the stimulation of a common antibiotic-mediated pathway. This interaction may be a mechanistic consequence of changes to cellular respiration observed in previous relevant research [8].

### 2.3. Translational Impact of MRF on Other Central Metabolic Pathways 

Various proteins were examined based on metabolic pathways of interest. Those significantly affected by the presence of MRF either alone or combined are listed in Table 2 (*p* ≤ 0.05). A list of the other key metabolic components that demonstrated the highest mean fold change following MRF supplementation is provided in the supplementary information (Tables S1 and S2). In addition, a heat map illustrating the influence of MRF and antibiotic treatment on the normalized peptide abundance of relevant proteins can be observed in the supplementary information (Figure S3).

Acetyl-coenzyme A (AcCoA) is a key molecule involved in central metabolism, allowing the replenishment of the NAD^+^-dependant TCA cycle and the glyoxylate shunt, leading to the generation of energy, and providing building blocks for the synthesis of essential compounds and secondary metabolites, including antibiotics [34]. MRF in combination with ampicillin treatment led to a significant positive fold change in the succinate-acetate/proton symporter (SATP) of AcCoA (×4.14) compared to the control culture (*p* ≤ 0.05) (Table 2). In addition, MRF alone produced a 0.41-fold change in PYKF, whilst treatment with MRF in combination with ampicillin led to a max positive fold change of ×0.68 compared to the control culture (*p* ≤ 0.05). Any rise in the peptide abundance of important proteins involved in the EMP pathway may be indicative of greater subsequent fluxes in downstream pathways [35]. Treatment with ampicillin alone did not lead to any significant shift in the normalized peptide abundance of proteins involved in AcCoA, the EMP pathways, or oxidative phosphorylation (OxPhos) (Table 2 and Table S2).

In terms of bacterial metabolic respiration, oxidative phosphorylation is key to central metabolism, as it generates ATP from ADP via an ATP synthase that utilizes proton motive force (PMF) by driving electrons through a membrane-bound electron transport system [34,36]. In the resistant species treated with ampicillin alone, the peptide abundance of proteins involved in oxidative phosphorylation was only significantly negatively affected compared to the control (*p* ≤ 0.05) (Table 2). Only supplementation with MRF either alone or combined with ampicillin treatment resulted in a positive fold change (Table 2). Membrane-bound ATP synthases (F0F1-ATPases) of bacteria are central to physiological function [37]. The fold changes in ATP synthases impacted by MRF supplementation are listed in Table 2. Koebmann et al. [38] demonstrated that an increase in ATPase activity led to the uncoupling of ATP activity. Studies have shown that the expression of ATP synthase α, β, and γ subunits and the interaction of β–γ subunits in *E. coli* may alter the growth of the bacterium [39,40]. Notably, a significant positive fold change in ATP synthases (subunits β, b, and γ) was observed when MRF was present alone or in combination with ampicillin (*p* ≤ 0.05) (Table 2). Therefore, these changes in the expression of key ATP synthases and/or changes in β–γ interaction central to physiological function may also be linked to the changes in resistant *E. coli* growth. In defense against oxidative stress, *E. coli* uses peroxide-scavenging enzymes, such as NADH peroxidase, alkyl hydroperoxide reductase (ahpCF), and the catalases KatG and KatE [41]. In this study, the significant overexpression of AHPF (×1.39) was observed in MRF cultures also treated with antibiotic (Table 2) (*p* ≤ 0.05). In addition, shifts in the expression patterns of the oxidoreductase subunits NUOB, NUOF, and NUOG were observed following MRF supplementation combined with/without antibiotic treatment (*p* ≤ 0.05) (Table 2).

The results showed that elevated levels of ROS produced by the resistant *E. coli* organism in response to the presence of MRF led to an overwhelming shift in the protective responses and defense systems of the organism, linking excessive endogenous ROS generation to cellular damage. Alternatively, ROS may not be the direct damaging agents, but initiate a regulated pathway for cell self-destruction [42]. Based on the data presented here, it is believed that a targeted surge of the TCA cycle or electron transport chain may lead to greater metabolic activity.

### 2.4. The Effect of MRF on NAD(H/^+^)

The NAD^+^/NADH ratio plays a pivotal role in regulating the intracellular redox state, metabolic redistribution, and microbial catabolism and is sensitive to the growth rate as well as other environmental factors [43]. A high NADH/NAD^+^ ratio triggers overflow metabolism [35], and a low ratio enhances glycolytic flux [44]. It is crucially important for continued cell growth that NADH be oxidized to NAD^+^ and a redox balance be achieved. Under aerobic growth, an increase in NADH availability influences metabolic distribution [45]. Many studies have characterized the manipulation of the NAD^+^/NADH ratio [43,46]. In this study, NAD(H/^+^) analysis revealed greater NAD(H/^+^) in resistant bacterial cultures supplemented with MRF (*p* ≤ 0.05) (Table 3). It is expected that the increase in bacterial respiration resultant from MRF supplementation converted NAD^+^ to NADH quicker than the control and ampicillin-only-containing cultures. Hence, the noted NADH increase in resistant bacterial cultures was proportionally higher than the NAD^+^ increase and resulted in a decreasing NAD^+^/NADH ratio (Table 3).

This is the first study to demonstrate cofactor modulation by prebiotic yeast material in a resistant organism, leading to metabolic changes affecting antibiotic susceptibility. The data demonstrated that NAD(H/^+^) may influence bacterial physiology and antibiotic susceptibility via hydroxyl radical formation resulting from TCA NADH generation.

### 2.5. The Effect of MRF Supplementation on Kinetic Growth of E. coli

**Figure 4:**
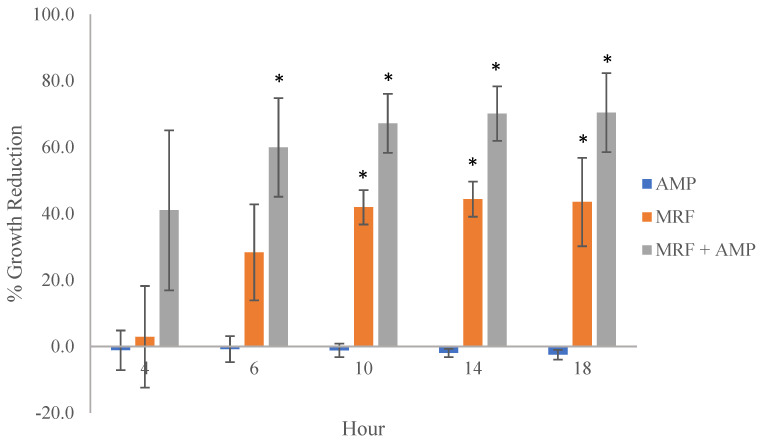
The effect of MRF and antibiotic treatment on the growth of antibiotic-resistant *E. coli* (*n* = 3) relevant to control culture. AMP (0.5 µgmL^−1^ AMP), MRF (5 mgmL^−1^), or a combination of AMP (0.5 mgmL^−1^) + MRF (5 mgmL^−1^) cultures. Standard error is represented by error bars. Means that were significantly different to one another within each time point are marked with an asterisk (*), *p* ≤ 0.05, ANOVA.

To demonstrate the impact of these findings on bactericidal drug resistance, we performed a kinetic growth analysis. Antibiotics induce changes in bacterial metabolism that promote the formation of ROS, which play a role in cell death [47]. We examined the growth of *E. coli* combined with exogenous MRF supplementation in liquid media treated with and without antibiotic treatment. The supplementation of *E. coli* resulted in the modification of growth over time (Figure 4). MRF reduced *E. coli* proliferation by 46% compared to antibiotic treatment; in addition, MRF and antibiotic treatment combined led to an adjunctive growth reduction of 73% compared to the antibiotic treatment (Figure 4).

MRF significantly reduced the growth of resistant *E. coli,* potentiating the antibiotic susceptibility of the resistant organism via functional metabolomic alterations involving changes in ROS production. The data demonstrated a link between ROS production, functional metabolic control, and the subsequent growth rate of the resistant organism.

## 3. Discussion

Yeast MRF was herein demonstrated to be a prebiotic capable of the endogenous modulation of ROS generation in *E. coli*. Notably, media supplemented with MRF resulted in a functional shift in central metabolic proteins in antibiotic-resistant *E. coli*. These results suggest that the greater peptide abundance of key TCA proteins in response to ROS overproduction characterizes the physiological changes observed in the resistant organism in response to MRF. Additionally, MRF may have contributed to the hyperactivation of the electron transport chain, with downstream implications for other relevant metabolic pathways. This study provides evidence that the observed changes effected by MRF have the potential to contribute to antibiotic-mediated efficacy and the associated accelerated respiration of *E. coli* [8]. It is believed that elevated levels of ROS produced in response to MRF in the growth culture overwhelmed the protective response of the organism, reverting it to a more antibiotic susceptible state. In addition, the greater proteomic response of the GS may have contributed to the greater tolerability and adaptation to oxidative stress. This may be a response to the induction of oxidative or antibiotic stress, or the overload of NADH compared to the control culture conditions. This represents the first report of yeast MRF enhancing phenotypic antibiotic susceptibility via metabolic perturbations and the proteomic modulation of key proteins central to respiration and metabolism. The data provided here demonstrated that exogenous supplementation with MRF may augment antibiotic mediated activity via endogenous ROS production. In addition, an adjuvant strategy to enhance antibiotic activity against resistant bacteria using a prebiotic yeast fraction was observed.

## 4. Materials and Methods

### 4.1. Bacteria and Yeast Cell Wall Preparation 

One Shot TOP10 chemically competent *E. coli* (Invitrogen™, Thermo Fisher Scientific, Dublin, Ireland) was used as the host strain for transformation. Recombinant *E. coli* transformed using pBR322 (Invitrogen™, Thermo Fisher Scientific, Ireland) was cultured in LB medium at 37 °C with the addition of 100 µgmL^−1^ of ampicillin (AMP) and 30 µgmL^−1^ tetracycline (TET). The recombinant strain was used as the resistant test organism throughout. Yeast mannan-rich fraction (MRF) from the cell wall of *S. cerevisiae* was provided by Alltech (Alltech Inc., Nicholasville, KY, USA). To ensure homogenous mixing, this suspension was then sonicated using an HTU Soni 130 ultrasonic processor at a power setting of 130 Watts for 3 min on ice. Aliquots were stored at 4 °C prior to use or at −70 °C for long-term storage. All antibiotics were obtained from Merck, Germany.

### 4.2. Inoculum Preparation and Storage

Frozen stocks of bacteria (5% (*v*/*v*)) in 70% glycerol were stored at −70 °C in LB broth (BD Difco™, Lennox, Fisher Scientific). Working plates were prepared by transferring a single colony using the spread plate technique to fresh agar and incubating overnight. Streaked plates were stored at 4 °C for up to six months. Fresh inoculum was prepared by isolating a single colony from working plates into approximately 25 mL of LB broth and grown overnight at 37 °C. Optical density (OD) at 595 nm was read using a UV-1601PC spectrophotometer (Shizmadzu Corporation, Kyoto, Japan).

### 4.3. Growth and Maintenance of Recombinant E. coli

LB medium (8 gL^−1^) was sterilized at 105 °C for 30 min. If agar was required, 15 gL^−1^ was added before autoclaving. To facilitate the growth of *E. coli*, the medium was cooled to approximately 55 °C, after which AMP and TET were added to a final concentration of 100 µgmL^−1^ and 30 µgmL^−1^, respectively. Agar plates were stored in the dark at 4 °C and were warmed to 37 °C prior to use.

### 4.4. ROS Quantification

A MAK-143 intracellular ROS kit (Merck, Germany) was used to measure the level of intracellular ROS according to manufacturer’s instructions. Overnight cultures of resistant *E. coli* were washed and diluted in fresh LB media (+/− MRF 0.01–0.5%, *w*/*v*) to an OD of 0.1 at λ_595nm_ and grown to ≥0.3 at 37 °C. ROS were induced by the addition of a master reaction mix (100 µL) to 80 µL of each treatment and incubation at 37 °C for 1 h. Following incubation, 20 µL of PBS, ampicillin (5 mgmL^−1^), or Menadione was added. Mean fluorescence intensity (MFI) was determined by a fluorescence microplate reader (Tecan Infinite M200 Pro microplate reader, Switzerland) (λex = 490nm/λem = 525nm). Initial relevant OD (background) was removed from each hourly measurement, respectively. Data are presented as means ± SD. Each measurement was performed in triplicate.

### 4.5. Preparation of Samples for Proteomic Analysis

Comparative proteomic analysis was performed with yeast *E. coli* control, yeast *E. coli* treated with 0.1 mgmL^−1^ AMP, yeast *E. coli* supplemented in MRF (1 mgmL^−1^) control, and yeast *E. coli* supplemented in MRF (1 mgmL^−1^) and treated with 0.1 mgmL^−1^ AMP (combination treatment). Each sample (100 µL) in duplicate (*n* = 8) was prepared using the iST kit (PreOmics, Munich, Germany) as per the manufacturer’s recommendations. Briefly, 50 µL of lysis buffer from the kit was added to each sample and heated at 95 °C at 1000 rpm for 10 min using a heating block. Samples were transferred to spin cartridges and subjected to tryptic digestion in a heating block set to 37 °C, 500 rpm for 2 h. Peptides were washed twice and then eluted by a centrifuge at 3800× *g* for 3 min. Peptides were dried and stored at −80 °C prior to LCMS analysis.

### 4.6. Proteomic Analysis

Reverse-phased capillary high-pressure liquid chromatography was conducted using a Thermo Scientific UltiMate™ 3000 nano RSLC system (Thermo Fisher Scientific, Basingstoke, UK) coupled directly in-line with a Thermo Orbitrap Fusion Tribrid Mass Spectrometer (Thermo Fisher Scientific, UK). The digested protein samples were resuspended in 50 μL of resuspension buffer containing 2% acetonitrile with 2% formic acid and sonicated in a water bath for 5 min. From each sample, 1 μL was loaded onto a C18 trapping cartridge (Thermo Scientific PepMap™ 100, C18, 300 μm × 5 mm) at a flow rate of 25 μL min^−1^ with 2% (*v*/*v*) acetonitrile (ACN) and 0.1% (*v*/*v*) trifluoroacetic acid (TFA) for 3 min before being resolved onto an C18 analytical column (Thermo Scientific Acclaim™ PepMap™ 100, 75 μm × 50 cm, 3 μm bead diameter column). Peptides were eluted using the following binary gradient: solvent A (0.1% (*v*/*v*) formic acid in LC–MS-grade water) and 2–27.5% solvent B (80% (*v*/*v*) ACN, 0.08% (*v*/*v*) formic acid in LC–MS-grade water) for 110 min at a flow rate of 300 nL min^−1^. For peptide ionization, a voltage of 1.8 kV was applied, and a capillary temperature of 320 °C was used.

Data-dependent acquisition with full scans in the 380–1500 m/z range was performed using the Orbitrap mass analyzer with a resolution of 120,000 (at m/z 200), a targeted automatic gain control (AGC) value of 4 × 10^5^, and a maximum injection time of 50 ms. The number of selected precursor ions for fragmentation was determined by the top-speed acquisition algorithm. Selected precursor ions were isolated in the Quadrupole with an isolation width of 1.6 Da. Peptides with a charge state of 2+ to 6+ were analyzed, and a dynamic exclusion was applied after 60 s. Precursor ions were fragmented using high-energy collision-induced dissociation (HCD) with a normalized collision energy of 28%, resulting MS/MS ions were measured in the linear ion trap. The typical MS/MS scan conditions were as follows: a targeted AGC value of 2 × 10^4^ and a maximum fill time of 35 min.

Quantitative label-free data analysis was performed using Progenesis QI for Proteomics (version 2.0; Nonlinear Dynamics, a Waters company, Newcastle upon Tyne, UK). Peptide and protein identification was achieved with Proteome Discoverer 2.2 using Sequest HT (Thermo Fisher Scientific, UK) and Percolator against an *E. coli* fasta database downloaded from Uniprot.org (containing 4050 reviewed sequences from Swiss-Prot), and the information was then imported into Progenesis QI software for further analysis. Only *p*-values ≤ 0.05 (ANOVA) and a minimum fold change of 1.5 were reported. A total of 1654 proteins were identified; of these, the proteins of interest were reported. To calculate the maximum fold change for a protein, Progenesis QI was used to determine the mean abundance for that protein under each set of experimental conditions. These mean values were then placed in a condition-vs-condition matrix to find the maximum (max) fold change between any two conditions’ mean protein abundances.

### 4.7. NAD(H/^+^) Determination

The NAD(H/^+^) ratio was determined using an NAD^+^/NADH Ratio assay kit (AssayGenie, Ireland). Overnight bacterial cultures were washed and diluted to 0.1 OD with relevant media: LB or LB combined with 1 mgmL^−1^ ampicillin (+/− MRF 0.5%, *w*/*v*). Bacteria were grown for 3–4 h at 37 °C. Following incubation, 1 mL was centrifuged at 14,000 rpm at 4 °C and suspended in cold PBS. Cells were spun again at 14,000 rpm and homogenized with either 100 μL NAD^+^ extraction buffer for NADH determination or 100 μL NADH extraction buffer for NAD^+^ determination. The suspension was heated at 60 °C for 5 min, and then 20 μL assay buffer and 100 μL of the opposite extraction buffer was added to neutralize the extracts. Following a brief vortex, the samples were spun at 14,000 rpm for 5 min. The supernatant was used for NAD^+^/NADH assays. For the preparation of a calibration curve, 10 mM NAD was made up by mixing 5 μL 1 mM standard solution and 495 μL distilled water. Aliquots of samples and standards (40 μL) were placed into wells of a clear flat-bottom 96-well plate. Fresh working reagent (80 μL) was prepared according to the manufacturer’s instructions and promptly added to each well. Optical density at time zero (OD0) and after a 15 min incubation period (OD15) was measured at 565 nm at room temperature. The NAD(H) concentration of the sample was calculated as follows: [NAD(H)] (µM^−1^) = ((ΔOD_SAMPLE_ − ΔOD_BLANK_)/Slope (µM^−1^)) ∗ n, where slope is the slope of the standard curve and n is the dilution factor.

### 4.8. Kinetic Growth Analysis

A kinetic growth study was performed to determine the effect of MRF on the growth of resistant *E. coli*. MRF (0.5%, *w*/*v*) was supplemented into the overnight growth media of both TOP10 non-transformed *E. coli* and *E. coli* transformed with pBR322 harboring resistance to ampicillin. Untreated bacterial cultures served as the negative control. A reference control with no antibiotic was included for each analysis. Replicate analysis was conducted using a Tecan Infinite M200 Pro microplate reader, Switzerland. The viable count was measured up to 18 h with defined measurement intervals, following a 5 s medium shake. Aliquots of 200 μL per well were analyzed in triplicate at OD λ_595nm_ in a sterile 96-well flat-bottom microtiter plate. Separately, aliquots of 5 µgmL^−1^ ampicillin were added per well (10 μL). To this, 20 μL aliquots of the test organism (adjusted to 0.01 OD at λ_595nm_ (approx. 1 × 10^−8^ cfu/mL)) taken from the exponential growth phase (overnight growth culture washed with fresh media via centrifuge (4000 rpm, 10 min) and grown for approx. 1–3 h) were added to each well. OD measurements were examined at 4, 6, 10, 14, and 18h across various test conditions to determine any modulation in growth compared to the control culture. Experiments were conducted using the same strain in identical media, and the experiment was repeated three times independently, with three replicates per sample.

### 4.9. Statistical Analysis

For ROS, NAD(H/^+^) determination, and growth analysis, one-way analysis of variance (ANOVA) and Fisher’s LSD multiple-comparisons tests were conducted to determine any significant differences among the means (Minitab statistical software package, version 20 (Coventry, UK)). Statistical significance between the normalized peptide abundance of resistant *E. coli* relative to the control was determined using ANOVA, T-test between pairs assuming unequal variance (EXCEL). Significant levels were defined using *p* ≤ 0.05.

## 5. Conclusions

It is evident from the relevant research and the data presented here that metabolism plays a significant role in oxidative stress and the phenotypic antibiotic susceptibility of *E. coli*. Previously, yeast MRF was noted to result in higher oxygen consumption, and, together with these results, we propose evidence of a link between cell physiology and antibiotic effectiveness, demonstrating a potential target for improved disease prevention strategies. In this study, MRF led to a cascade of modulations in the central metabolic pathways, bacterial growth, and stress response, that together contributed to the potentiation of phenotypic resistance or physiological antibiotic susceptibility. Enhancing ROS production or interfering with protection against ROS via MRF supplementation represents a novel strategy for potentially augmenting antibiotic treatment. With the alarming increase in antibiotic resistance in bacteria, a better understanding of the potential exogenous influences that could enhance antibiotic efficacy would support future strategies to combat resistance. Increasing our knowledge of the interaction between prebiotics such as MRF and resistant microorganisms may make it possible to reduce antibiotic use whilst promoting animal health strategically and naturally.

## Figures and Tables

**Table 1:** The influence of MRF (1 mgmL^−1^) and antibiotic treatment AMP (0.1 mgmL^−1^ AMP) on fold-change expression (normalized peptide abundance) of intermediates of the citric acid cycle of antibiotic-resistant *E. coli* relative to the control.

Citric Acid Cycle						MRF		AMP		MRF + AMP		Highest Mean Condition
Accession	Protein	PC	UP	PSM	SC (%)	Fold Change ^a^	*p*-Value	Fold Change ^a^	*p*-Value	Fold Change ^a^	*p*-Value	
P0A9Q3	ARCA	4	3	41	27	+0.52	0.19	−0.01	0.92	+0.78	0.03	MRF + AMP
Q8X722	ICD	8	8	62	23	−0.20	0.30	−0.13	0.40	+0.72	0.03	MRF + AMP
A0A0H3JCM9	GLTA	2	2	17	6	+0.37	0.22	+0.46	0.17	+1.65	0.05	MRF + AMP
Q8X957	ACNB	9	9	127	21	+0.04	0.73	−0.01	0.92	+0.62	0.02	MRF + AMP
P0AFG5	SUCA	3	3	40	7	+0.27	0.14	+0.65	0.06	+1.59	0.00	MRF + AMP
Q8XDQ0	FRDA	6	6	47	12	+0.26	0.18	−0.12	0.52	+0.83	0.01	MRF + AMP
Q8X609	ACEB	3	3	43	19	−0.26	0.10	−0.51	0.01	+0.15	0.16	MRF +AMP
P61891	MDH	3	2	23	21	+0.60	0.02	−0.05	0.54	+0.54	0.02	MRF
Q8XDS0	ASPA	4	4	31	12	−0.27	0.23	−0.31	0.15	+1.02	0.06	MRF + AMP
Q8X743	PPC	6	6	108	18	+0.52	0.17	+0.31	0.21	+0.45	0.12	MRF

^a^ Fold-change increase symbolized with a +. Fold-change decrease symbolized with a −. Unequal variances T-test comparison of each treatment group to control was performed, ANOVA (*p* ≤ 0.05). Abbreviations: PC, peptide count; UP, unique peptides; SC, sequence coverage (%); PSM, peptide spectrum match; ARCA, aerobic respiration control protein; ICD, isocitrate dehydrogenase; GLTA, citrate synthase; ACNB, aconitate hydratase B; SUCA, 2-oxoglutarate dehydrogenase E1 component; FRDA, fumarate reductase flavoprotein subunit; ACEB, malate synthase; MDH, malate dehydrogenase.

**Table 2:** The influence of MRF (1 mgmL^−1^) and antibiotic treatment (0.1 mgmL^−1^ AMP) on fold-change expression (normalized peptide abundance) of key components of central metabolic pathways relative to the control.

	Other Metabolic Pathways					MRF		AMP		MRF + AMP		Highest Mean Condition
Accession	Protein	PC	UP	PSM	SC (%)	Fold Change ^a^	*p*-Value	Fold Change ^a^	*p*-Value	Fold Change ^a^	*p*-Value	
	Acetyl-coenzyme A											
P0AC99	SATP	1	1	3	5	0.03	0.92	0.88	0.18	4.14	0.04	MRF + AMP
	EMP Pathway											
P0A860	TPIA	2	2	15	5	3.20	0.01	−0.12	0.61	3.08	0.08	MRF
P62709	GPMA	7	7	39	31	0.93	0.04	0.16	0.50	0.4	0.19	MRF
Q8XDE9	GPMI	9	9	78	24	0.85	0.05	0.16	0.09	1.09	0.08	MRF + AMP
P0AD62	PYKF	12	12	79	27	0.41	0.03	0.06	0.52	0.68	0.00	MRF + AMP
Q8X7H3	FBAB	5	5	35	21	0.91	0.02	−0.30	0.14	0.3	0.14	MRF
Q8XCF0	FBP	4	4	23	17	0.69	0.02	0.13	0.05	0.92	0.14	MRF + AMP
Q8X926	PGL	3	3	12	15	0.64	0.11	0.3	0.13	0.79	0.03	MRF + AMP
P0AB69	PNTB	3	3	15	7	0.20	0.34	0.03	0.84	1.27	0.03	MRF + AMP
P0A797	PFKA	7	7	36	20	1.17	0.02	0.38	0.08	1.86	0.16	MRF + AMP
Q8XE22	PFKB	3	3	17	12	1.08	0.02	−0.19	0.13	0.34	0.35	MRF
Q8XDE9	GPMI	9	9	78	24	0.85	0.05	0.16	0.09	1.09	0.08	MRF + AMP
	OxPhos Pathway											
P0AFC9	NUOB	2	2	12	6	1.41	0.01	1.33	0.09	4.43	0.09	MRF + AMP
Q8XCX1	NUOF	2	2	11	5	−0.44	0.03	−0.19	0.03	0.3	0.03	MRF + AMP
Q8XCX2	NUOG	7	7	34	9	0.03	0.83	0.07	0.91	1.65	0.03	MRF + AMP
P0ABJ0	CYOB	1	1	7	1	−0.70	0.06	−0.57	0.02	0.01	0.86	MRF + AMP
P58646	ATPC	3	3	39	23	0.25	0.53	−0.74	0.03	−0.54	0.15	MRF
P0ABA8	ATPG	7	7	55	23	−0.17	0.33	−0.17	0.33	1.21	0.01	MRF + AMP
P0ABB2	ATPA	9	8	62	27	−0.06	0.46	−0.36	0.02	0.2	0.30	MRF + AMP
P0ABA2	ATPF	3	3	14	6	2.03	0.06	−0.05	0.02	2.03	0.00	MRF
P0ABB6	ATPD	14	14	104	40	0.14	0.02	−0.12	0.01	1.17	0.16	MRF + AMP
P0A7G8	RECA	3	3	14	6	8.70	0.06	1.58	0.06	1.72	0.00	MRF
	Stress and Signaling											
P0AGD2	SODC	4	4	31	28	1.07	0.83	1.03	0.91	2.65	0.03	MRF + AMP
Q8XBT4	AHPF	9	9	49	17	0.35	0.07	0.09	0.50	1.39	0.01	MRF + AMP
P0ACG0	H-NS	5	5	13	28	1.20	0.21	0.35	0.12	1.15	0.02	MRF
P0ACJ2	LRP	4	4	19	12	0.56	0.05	0.31	0.06	0.93	0.10	MRF + AMP
P58162	DSBD	1	1	8	3	2.69	0.00	0.9	0.01	2.79	0.03	MRF + AMP
P58320	DSBG	3	3	29	14	0.68	0.02	−0.03	0.69	1.21	0.09	MRF + AMP
P0C0V1	DEGP	5	5	30	12	0.15	0.18	0.29	0.05	0.48	0.05	MRF + AMP
Q8XE55	PPID	15	15	95	26	0.26	0.30	−0.23	0.05	0.77	0.00	MRF + AMP

^a^ Fold-change increase symbolized with a +. Fold-change decrease symbolized with a −. Unequal variances T-test comparison of each treatment group to control was performed, ANOVA (*p* ≤ 0.05). Abbreviations: PC, peptide count; UP, unique peptides; SC, sequence coverage (%); PSM, peptide spectrum match; SATP, succinate-acetate/proton symporter; TPIA, triosephosphate isomerase; GPMA, 2,3-bisphosphoglycerate-dependent phosphoglycerate mutase; GPMI, 2,3-bisphosphoglycerate-independent phosphoglycerate mutase; PYKF, pyruvate kinase I; FBAB, fructose-bisphosphate aldolase class; FBP, fructose-1,6-bisphosphatase class 1; PGL, 6-phosphogluconolactonase; PNTB, NAD(P) transhydrogenase subunit beta; PFKA, ATP-dependent 6-phosphofructokinase isozyme 1; PFKB, phosphofructokinase; GPMI, 2,3-bisphosphoglycerate-independent phosphoglycerate mutase; NUOB, NADH-quinone oxidoreductase subunit B; NUOF, NADH-quinone oxidoreductase subunit F; NUOG, NADH-quinone oxidoreductase subunit G; CYOB, cytochrome bo(3) ubiquinol oxidase subunit 1; ATPC, ATP synthase ε chain; ATPG, ATP synthase γ chain; ATPA, ATP synthase subunit α; ATPF, ATP synthase subunit b; ATPD, ATP synthase subunit β; RECA, protein RecA; SODC, superoxide dismutase (Cu-Zn); AHPF, alkyl hydroperoxide reductase; H-NS, DNA-binding protein; LRP, leucine-responsive regulatory protein; DSBD, thiol:disulfide interchange protein; DSBG, thiol:disulfide interchange protein; DEGP, periplasmic serine endoprotease; PPID, peptidylprolyl isomerase.

**Table 3:** Metabolic fluxes in NAD(H/^+^) concentrations (µM).

	Control	AMP	MRF	AMP + MRF
NADH	1.720 ± 0.266	1.758 ± 0.119	2.700 ± 0.176 ^ab^	2.553 ± 0.201 ^ab^
NAD^+^	5.095 ± 0.022	4.910 ± 0.336	6.280 ± 0.004 ^ab^	6.387 ± 0.312 ^ab^
NADH/NAD^+^	0.340 ± 0.051	0.358 ± 0.039	0.427 ± 0.028 ^a^	0.397 ± 0.026
NAD^+^/NADH	2.983 ± 0.418	2.820 ± 0.292	2.352 ± 0.151	2.524 ± 0.161
Total NAD(H/^+^)	6.82	6.67	8.98 ^ab^	8.94 ^ab^
% Change in total NAD(H/^+^)	-	−2.2	31.8	31.2

Percentage change in total NAD(H/^+^) was based on the concentration of the control experiments. Values marked with an ‘a’ represent values that were significantly different to the relevant control culture. Values marked with a ‘b’ represent values that were significantly different to the relevant AMP-containing culture (Dunnett multiple-comparison, ANOVA (*p* ≤ 0.05)).

## Data Availability

Not applicable.

## Supplementary Materials

The following supporting information can be downloaded at: https://www.mdpi.com/article/10.3390/ijms24010218/s1.
