# Supplementary material for: Yeast Mannan-Rich Fraction Modulates Endogenous Reactive Oxygen Species Generation and Antibiotic Sensitivity in Resistant E. coli
Source: Int J Mol Sci. 2022 Dec 22;24(1):218. doi: 10.3390/ijms24010218 (PMC9820725; doi:10.3390/ijms24010218)
Supplement: Supplementary file 1 [file ijms-24-00218-s001.zip › ijms-2059713-supplementary.pdf]

Supplementary Information (SI)

This file contains supplementary information for manuscript entitled "Yeast mannan-rich fraction modulates endogenous reactive oxygen species generation and antibiotic sensitivity in resistant *E. coli*".

Smith H.*,

Helen Smith*
Email: hsmith@alltech.com

This file includes:

Figures S1, S2 and S3
Tables S1 and S2

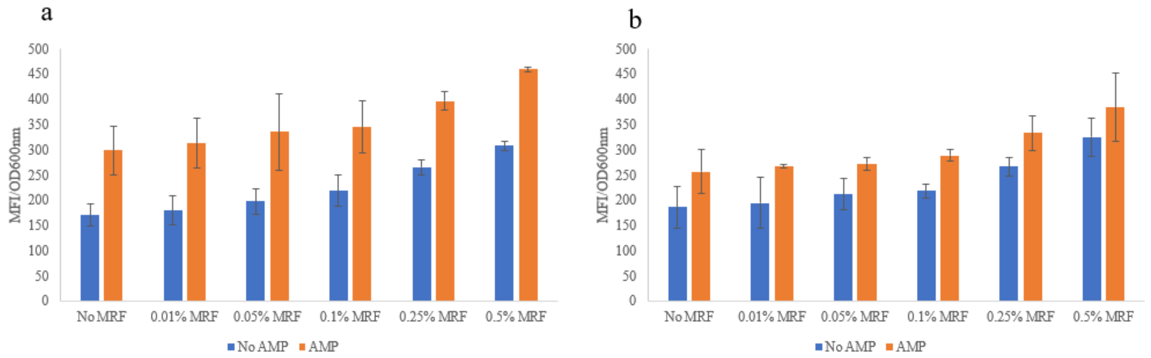

Figure S1. The effect of MRF (0.01 – 0.5%) on ROS (MFI) combined with and without ampicillin. Diagram displays mean fluorescent intensity (MFI/OD600nm) of [a] AMP susceptible *E. coli* treated with AMP (5µgmL^{-1}) compared to no antibiotic treatment and [b] AMP resistant *E. coli* treated with AMP (5mgmL^{-1}) compared to no antibiotic treatment. Standard deviation is represented by error bars (n=6).

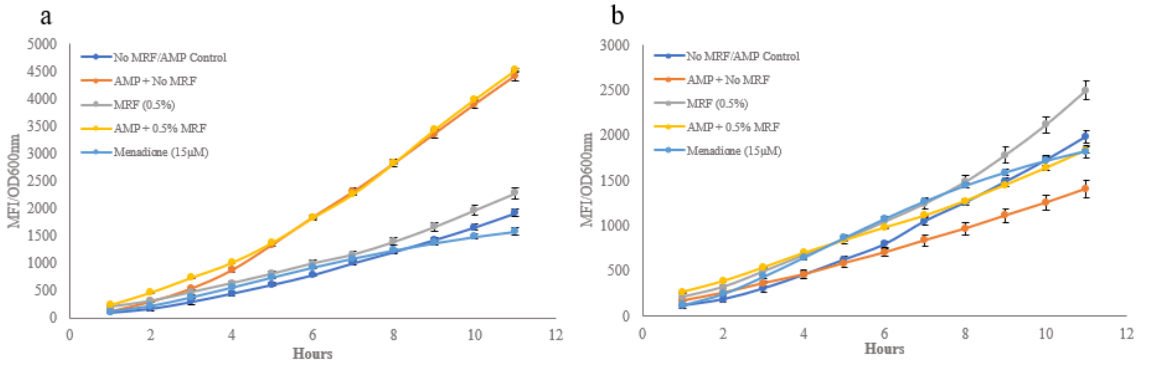

Figure S2. The effect of MRF on ROS (MFI) with and without ampicillin treatment, over time. Diagram displays mean fluorescent intensity (MFI/OD600nm) of [a] AMP susceptible *E. coli* over time; control (No MRF/AMP)] compared to AMP (5µgmL^{-1}), MRF (0.5%, w/v) and MRF (0.5%, w/v) combined with AMP (5µgmL^{-1}); [b] AMP resistant *E. coli* over time; control (No MRF/AMP) compared to AMP (5mgmL^{-1}), MRF (0.5%, w/v) and MRF (0.5%, w/v) combined with AMP (5mgmL^{-1}). Standard deviation is represented by error bars (n=6). Means that are significantly different to the respective control conditions are marked with an asterisk [*] ($p \leq 0.05$, ANOVA, Fisher-LSD).

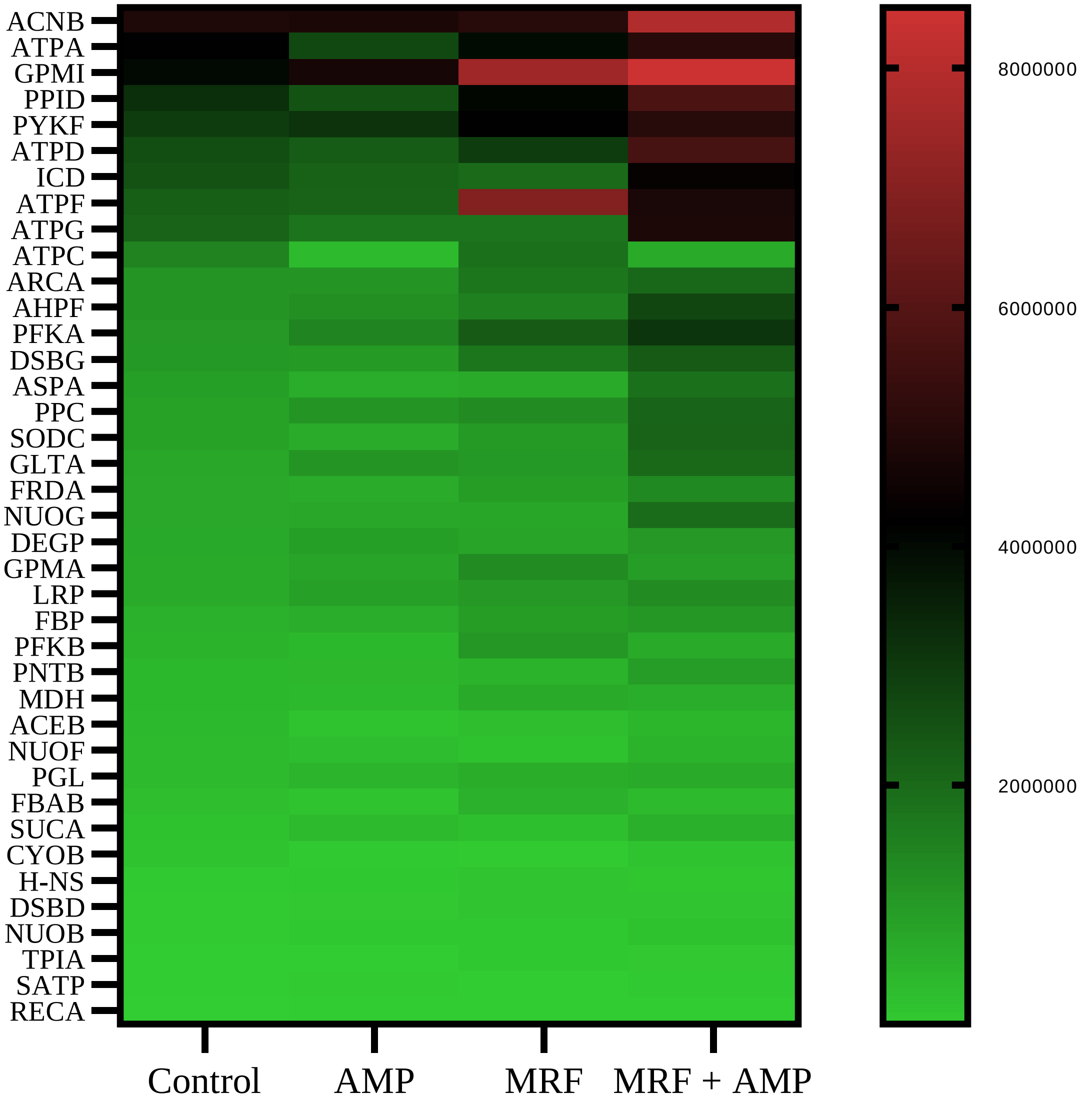

Figure S3. The influence of MRF and antibiotic treatment on normalized peptide abundance of relevant proteins of interest. Control culture treated with Amp (0.1 mgmL^{-1} Amp), MRF (0.1%, w/v), or combination of Amp (0.1mgmL^{-1}) + MRF (0.1%, w/v).

Table S1. The influence of MRF (1 mgmL^{-1}) and antibiotic treatment (0.1 mgmL^{-1} AMP) on fold change expression (normalized peptide abundance) of other detected intermediates of the citric acid cycle compared to control.

Accession	Protein	PC	UP	PSM	SC (%)	MRF Fold change[a]	MRF *p*-value	AMP Fold change[a]	AMP *p*-value	MRF + AMP Fold change[a]	MRF + AMP *p*-value	Highest Mean Condition
Q8X7C7	ACNA	9	9	71	12	-0.15	0.14	-0.21	0.09	+0.44	0.28	MRF + AMP
P0A838	SUCC	8	8	73	24	+0.43	0.20	-0.18	0.21	+0.17	0.21	MRF
P0AGF1	SUCD	6	6	48	24	+0.09	0.56	-0.40	0.17	-0.57	0.12	MRF
P0AC43	SDHA	2	2	12	3	+0.55	0.10	+0.22	0.40	+0.19	0.55	MRF
A0A0H3JRL7	FUMA	4	2	10	10	+2.06	0.25	+1.61	0.30	+3.96	0.08	MRF + AMP
P0A8Q2	FRDC	1	1	6	8	+0.45	0.28	+0.09	0.71	-0.32	0.29	MRF
Q8XDS0	ASPA	7	7	31	12	-0.27	0.23	-0.31	0.15	+1.02	0.06	MRF + AMP
Q8X743	PPC	14	14	102	18	+0.52	0.17	+0.31	0.21	+0.45	0.12	MRF

[a]Fold change increase symbolized with a [+]. Fold change decrease symbolized with a [-]. Unequal variances T-test comparison of each treatment group to control was performed, ANOVA ($p \leq 0.05$). Abbreviations: PC; Peptide count, UP; Unique peptides, SC (%); PSM; Peptide spectrum match; Sequence coverage (%); ACNA; Aconitate hydratase, SUCC; Succinate--CoA ligase [ADP-forming] subunit beta, SUCD; Succinate-CoA ligase [ADP-forming] subunit alpha, SDHA; Succinate dehydrogenase flavoprotein subunit, FUMA; Fumarate hydratase class I, FRDC; Fumarate reductase subunit C, ASPA; Aspartate ammonia-lyase, PPC; Phosphoenolpyruvate carboxylase.

Table S2. The influence of MRF (1 mgmL^{-1}) and antibiotic treatment (0.1 mgmL^{-1} AMP) on fold change expression (normalized peptide abundance) of other detected key components of central metabolic pathways compared to control.

							MRF		AMP		MRF + AMP		
	Accession	Protein	PC	UP	PSM	SC (%)	Fold change[a]	*p*-value	Fold change[a]	*p*-value	Fold change[a]	*p*-value	Highest Mean Condition
Acetyl-coenzyme A	Q8XCU8	PTA	10	10	56	18	+0.12	0.62	-0.25	0.39	+0.32	0.23	MRF + AMP
	P0A6A5	ACKA	2	2	9	8	+0.19	0.52	+0.21	0.51	+0.81	0.35	MRF + AMP
	Q8X5T5	ACS	1	1	3	2	-0.42	0.27	-0.17	0.54	+0.52	0.23	MRF + AMP
	Q8X6L4	POXB	6	6	31	10	+0.04	0.75	+0.01	0.94	+0.57	0.09	MRF + AMP
EMP pathway	P0A8A6	PPSR	1	1	4	3	-0.24	0.12	-0.17	0.10	+0.27	0.33	MRF + AMP
	P0A9B4	GAPA	9	9	70	30	+0.95	0.07	+0.05	0.82	+0.09	0.68	MRF
	Q8XD03	PGK	11	11	67	26	-0.01	0.86	-0.12	0.35	+1.27	0.06	MRF + AMP
	P0A6Q1	ENO	13	13	71	46	+3.62	0.12	+0.30	0.36	+3.92	0.06	MRF + AMP
	P0ADF7	EDD	2	2	10	7	+0.91	0.18	-0.18	0.62	+0.47	0.33	MRF
	Q8X774	PNTA	1	1	4	2	-0.44	0.06	-0.25	0.17	+0.34	0.17	MRF + AMP
	Q7DBF8	GND	9	9	61	28	+0.28	0.24	-0.18	0.37	+0.65	0.23	MRF + AMP
	P0A8A6	PPSR	1	1	4	3	-0.24	0.12	-0.17	0.11	+0.27	0.33	MRF + AMP
OxPhos	P0ABJ0	CYOB	1	1	7	1	-0.70	0.06	-0.57	0.02	+0.01	0.86	MRF + AMP
Bacterial stress signalling and response	P66828	SODA	4	4	46	18	+0.17	0.47	-0.31	0.33	+0.28	0.54	MRF + AMP
	P0AGD5	SODB	1	1	5	8	+0.81	0.39	+0.42	0.61	+0.81	0.36	MRF + AMP
	A0A0H3JFP5	KATE	18	18	110	24	+0.08	0.86	-0.29	0.56	+0.32	0.51	MRF + AMP
	Q7A978	KATG1	10	10	53	13	+0.66	0.09	+0.05	0.55	+0.35	0.45	MRF
	Q7ABA5	RPOS	1	1	11	3	+0.07	0.36	+0.33	0.35	+0.79	0.10	MRF + AMP
	P0ACK0	CRP	6	6	38	33	+0.39	0.12	-0.40	0.16	+0.05	0.81	MRF
	P0AE89	CPXR	3	3	33	13	+0.06	0.75	-0.49	0.13	-0.29	0.22	MRF
	P66798	UVRY	1	1	7	6	+0.43	0.26	-0.20	0.46	+0.87	0.06	MRF + AMP
	Q8XDH7	TREA	7	7	59	14	-0.26	0.18	-0.21	0.22	+0.09	0.44	MRF + AMP
	P0A6Y3	IHF	1	1	6	10	+1.32	0.15	-0.40	0.19	+1.77	0.16	MRF + AMP
	P0A974	CSPE	2	2	6	39	-0.33	0.13	-0.11	0.63	+0.10	0.56	MRF + AMP
	P0AEG5	DSBA	4	4	23	24	+0.55	0.24	-0.01	0.96	-0.58	0.22	MRF
	P0AEG7	DSBC	2	2	14	7	+0.51	0.49	+0.65	0.41	+3.63	0.17	MRF + AMP
	P0AFL5	PPIA	2	2	18	15	+0.07	0.27	+0.01	0.77	+1.71	0.20	MRF + AMP

[a]Fold change increase symbolized with a [+]. Fold change decrease symbolized with a [-]. Unequal variances T-test comparison of each treatment group to control was performed, ANOVA ($p \leq 0.05$). Abbreviations: PC; Peptide count, UP; Unique peptides, SC (%); PSM; Peptide spectrum match; Sequence coverage (%); PTA; Phosphate acetyltransferase, ACKA; Acetate kinase, ACS; Acetyl-coenzyme A synthetase, POXB; Pyruvate dehydrogenase, PPSR; Phosphoenolpyruvate synthase regulatory protein, GAPA; Glyceraldehyde-3-phosphate dehydrogenase A, PGK; Phosphoglycerate kinase, ENO; Enolase, EDD; Phosphogluconate dehydratase, PNTA; NAD(P) transhydrogenase subunit alpha, GND; 6-phosphogluconate dehydrogenase, decarboxylating, PPSR; Phosphoenolpyruvate synthase regulatory protein, CYOB; Cytochrome bo(3) ubiquinol oxidase subunit 1, SODA; Superoxide dismutase [Mn], SODB; Superoxide dismutase [Fe], KATE; Catalase, KATG1; Catalase-peroxidase 1, RPOS; RNA polymerase sigma factor RpoS, CRP; cAMP-activated global transcriptional regulator, CPXR; Transcriptional regulatory protein, UVRY; Response regulator, TREA; Putative periplasmic trehalase, IHF; Integration host factor subunit beta, CSPE; Cold shock-like protein, DSBA; Thiol:disulfide interchange protein, DSBC; Thiol:disulfide interchange protein, PPIA; Peptidyl-prolyl cis-trans isomerase A.
